# Supplementary figures and images for: Investigation of mobile genetic elements and their association with antibiotic resistance genes in clinical pathogens worldwide
Source: PLoS One. 2025 Aug 18;20(8):e0330304. doi: 10.1371/journal.pone.0330304 (PMC12360581; doi:10.1371/journal.pone.0330304)

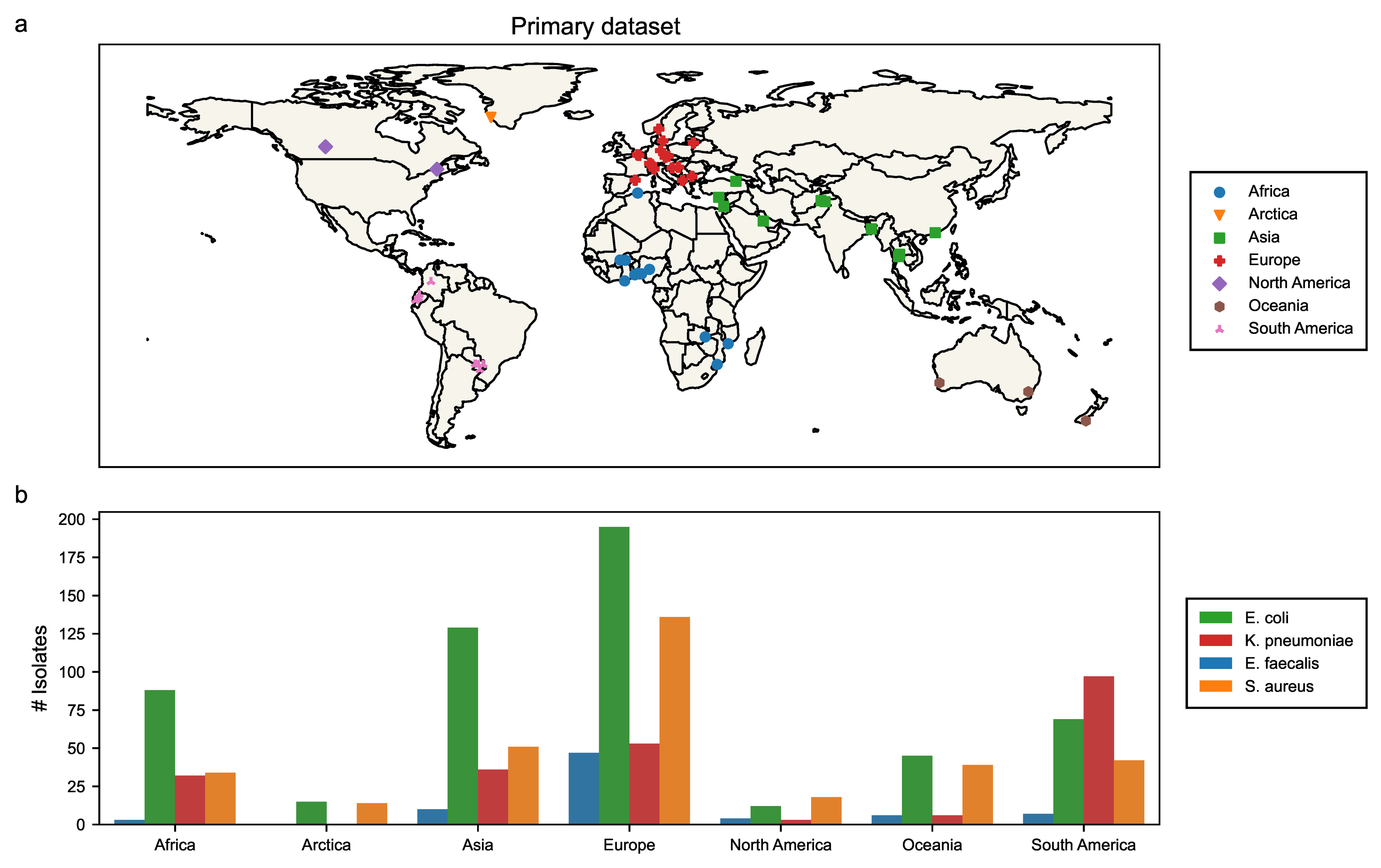

Supplement: S1 Fig — Rare sequence types for a given species (frequency < 5%) were amalgamated into the other category. The rare sequence type constituted ~32%−80%. Map was created using shapefiles from Natural Earth. (TIF) [file pone.0330304.s005.tif]

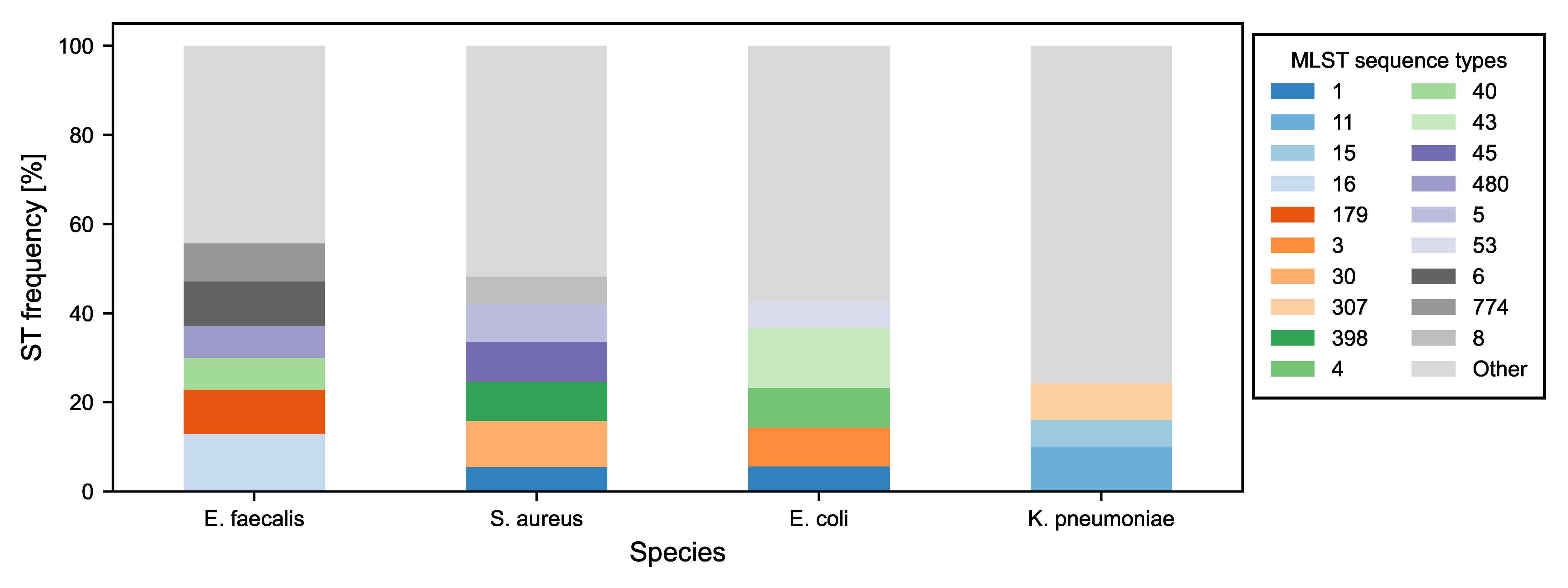

Supplement: S2 Fig — a) Locations where the bacterial isolates were collected. The marker shape and colour designate the geographical region.; b) barplot showing the number of isolates per bacterial species and region. (TIF) [file pone.0330304.s006.tif]

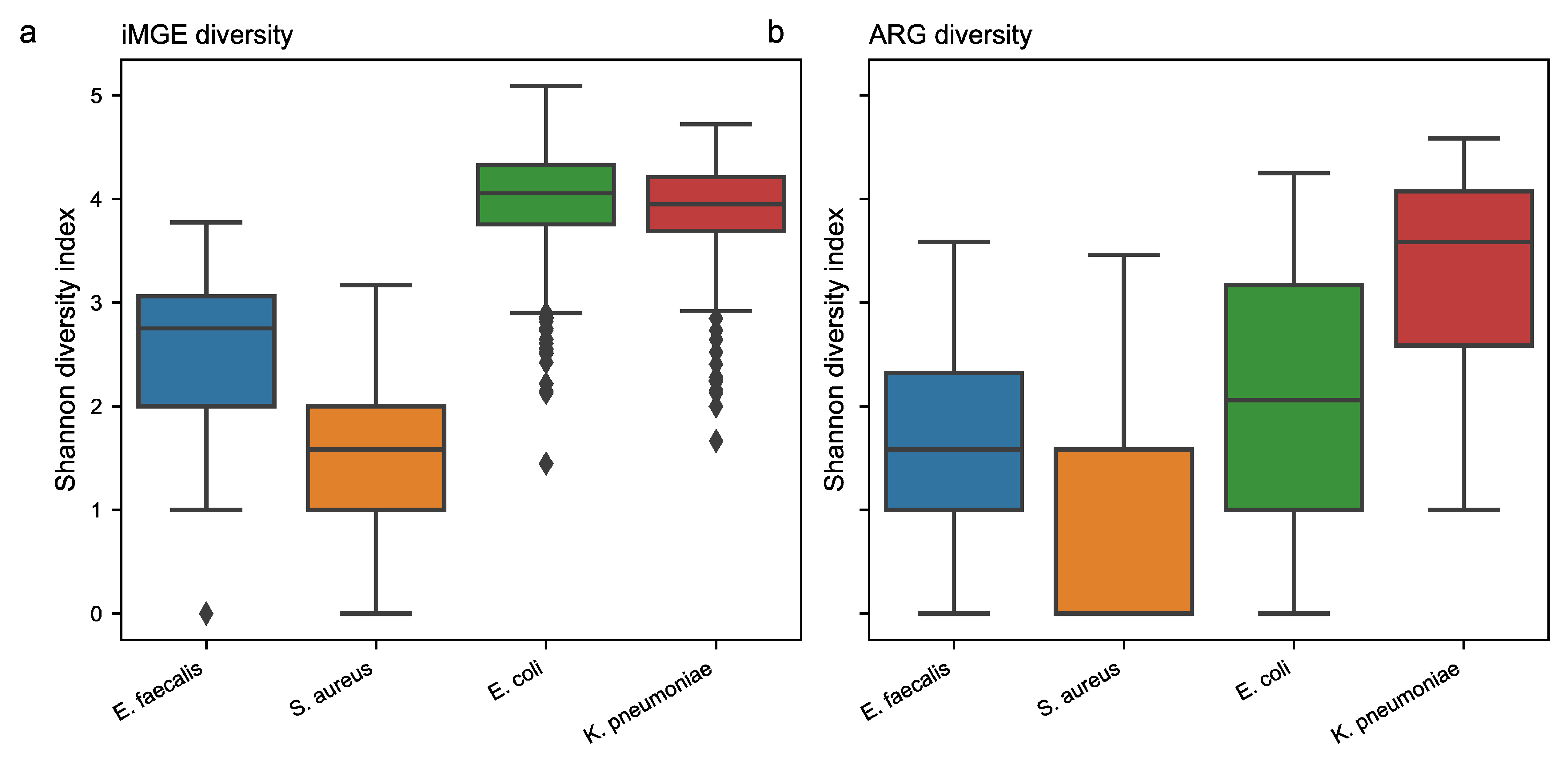

Supplement: S3 Fig — (TIF) [file pone.0330304.s007.tif]

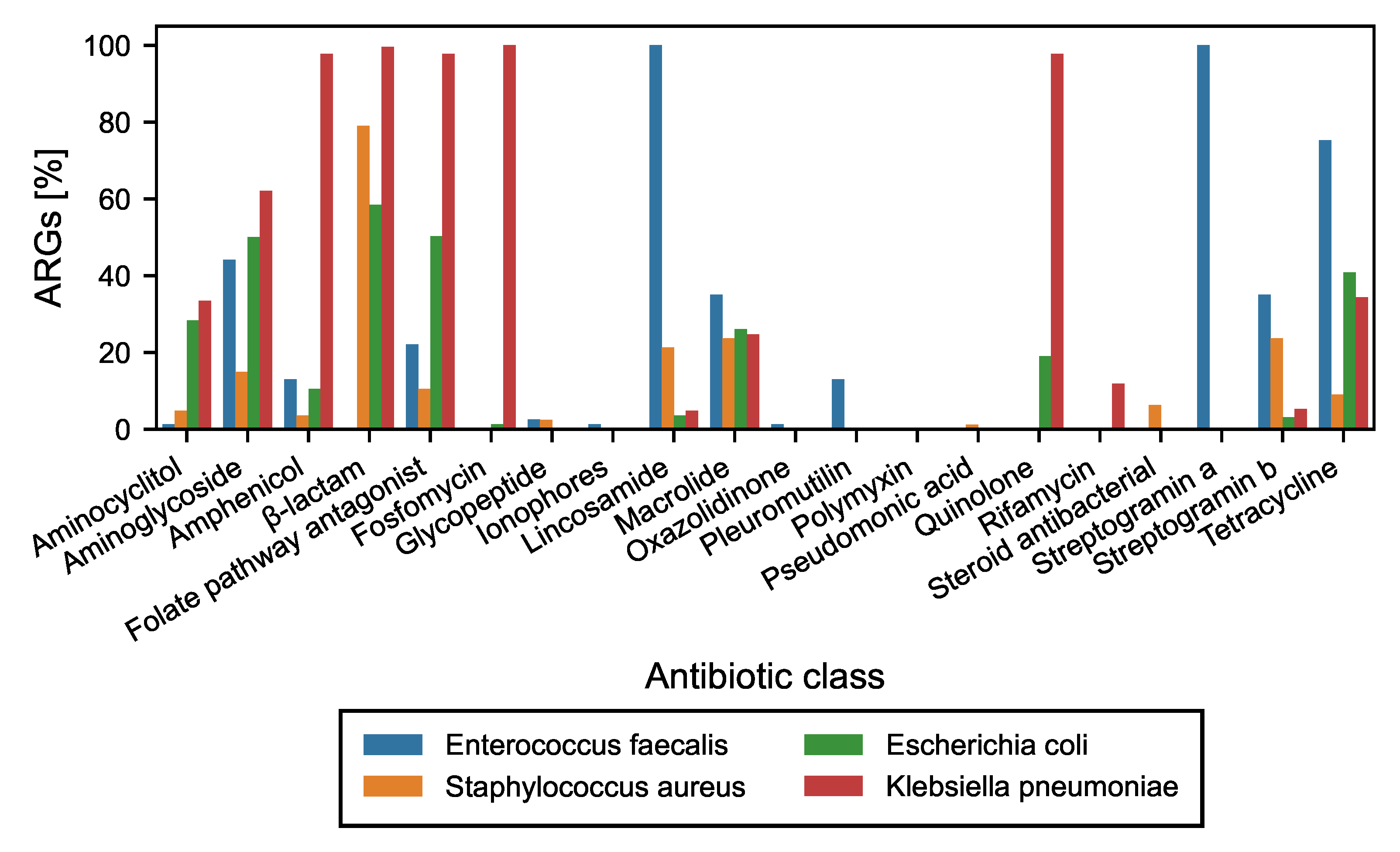

Supplement: S4 Fig — (TIF) [file pone.0330304.s008.tif]

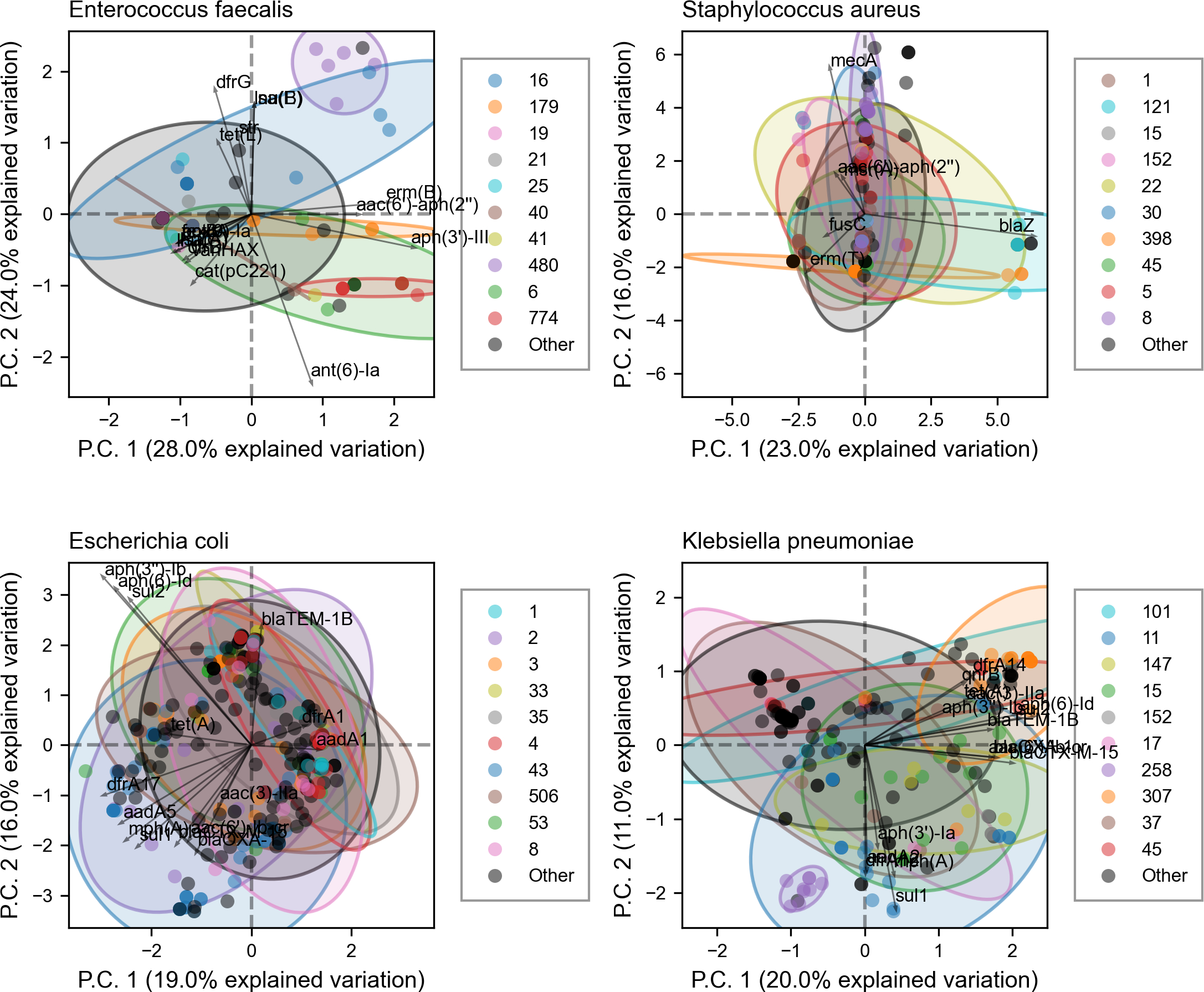

Supplement: S5 Fig — The scores are colored by MLST ST. The ten most common STs are displayed, and the rest are amalgamated into the other category. Ellipses shows the 90% confidence of score position. (TIF) [file pone.0330304.s009.tif]

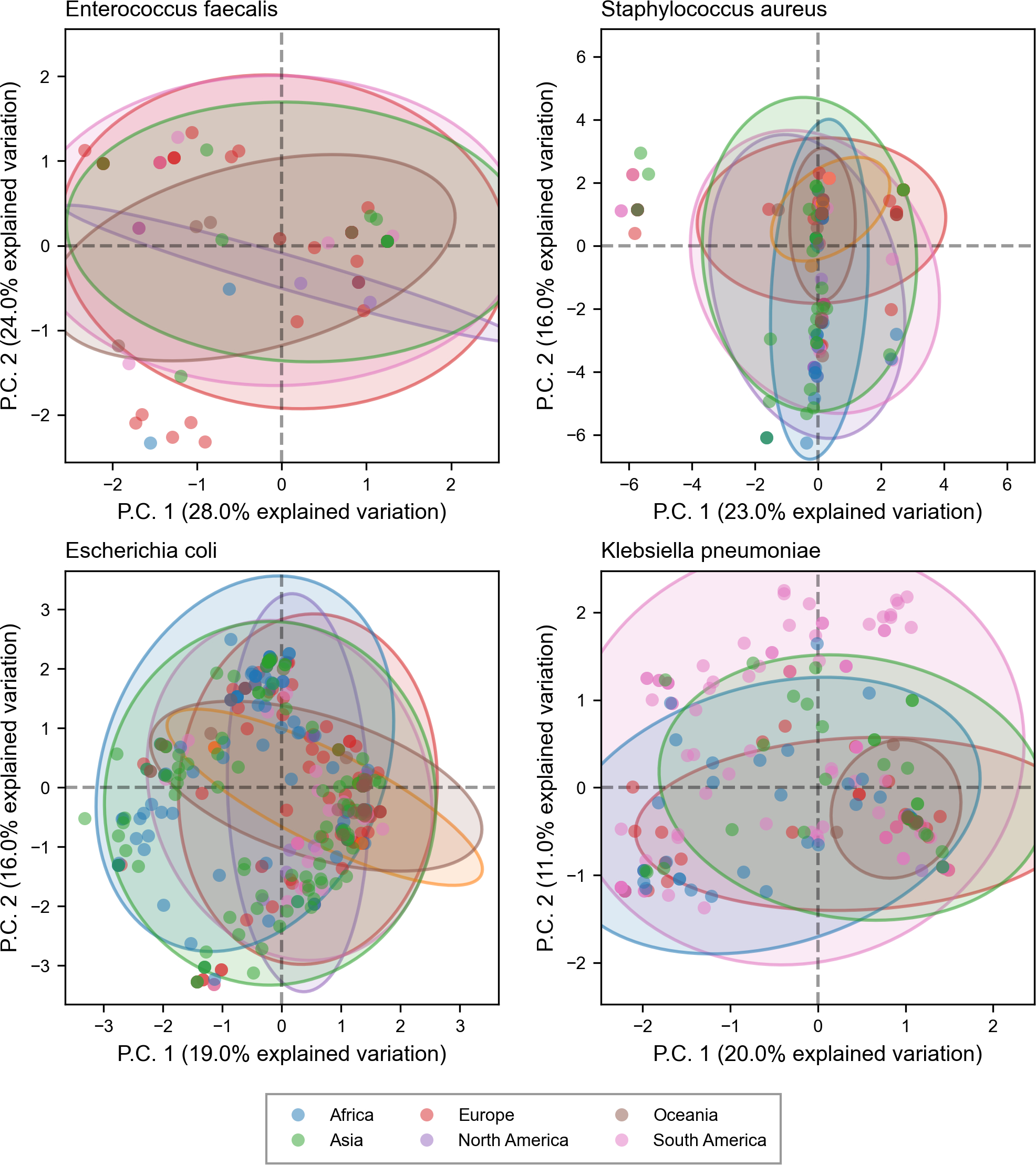

Supplement: S6 Fig — Ellipses show the 90% confidence of score position. (TIF) [file pone.0330304.s010.tif]

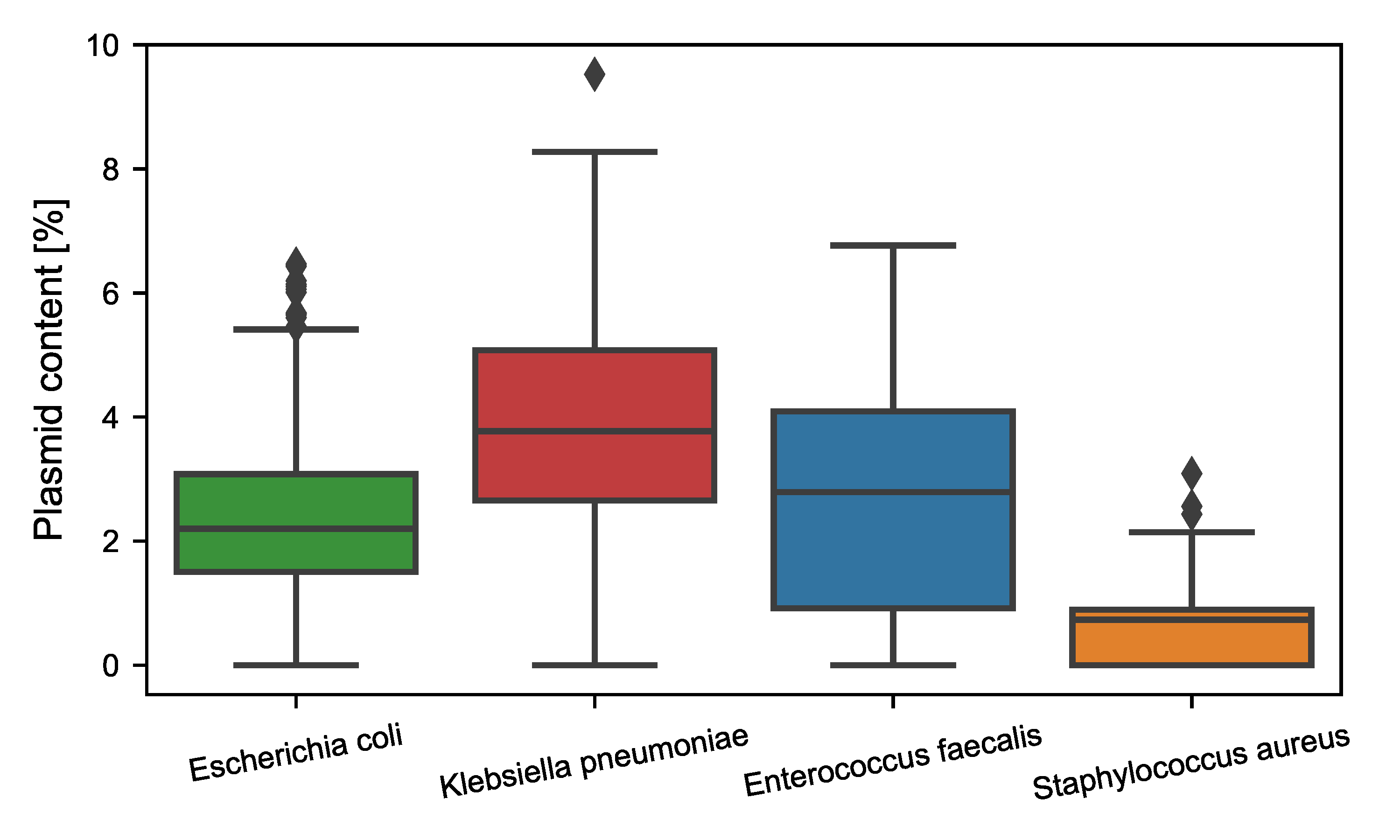

Supplement: S7 Fig — (TIF) [file pone.0330304.s011.tif]

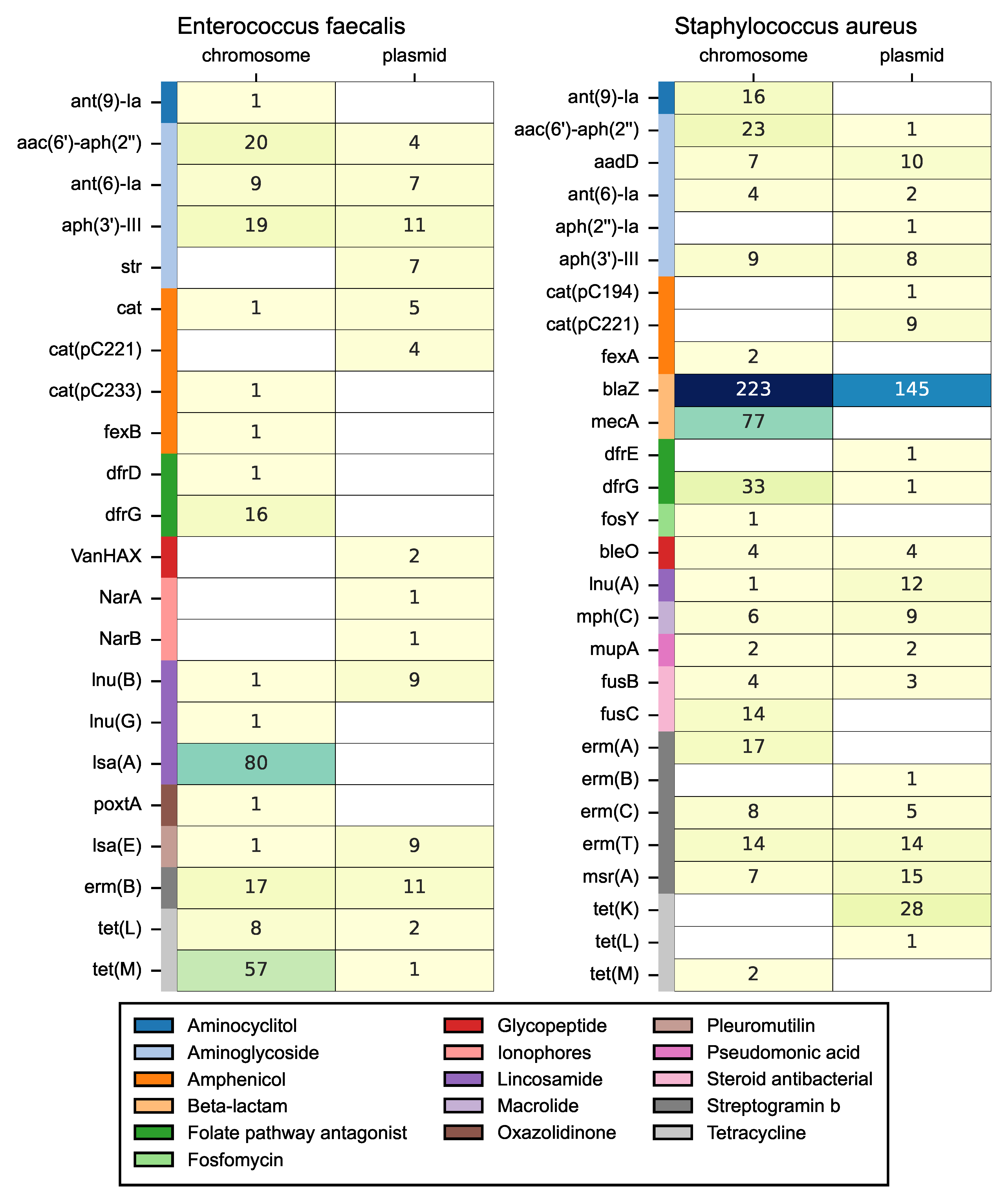

Supplement: S8 Fig — Genes are coloured by the class of antibiotics they yield resistance to. (TIF) [file pone.0330304.s012.tif]

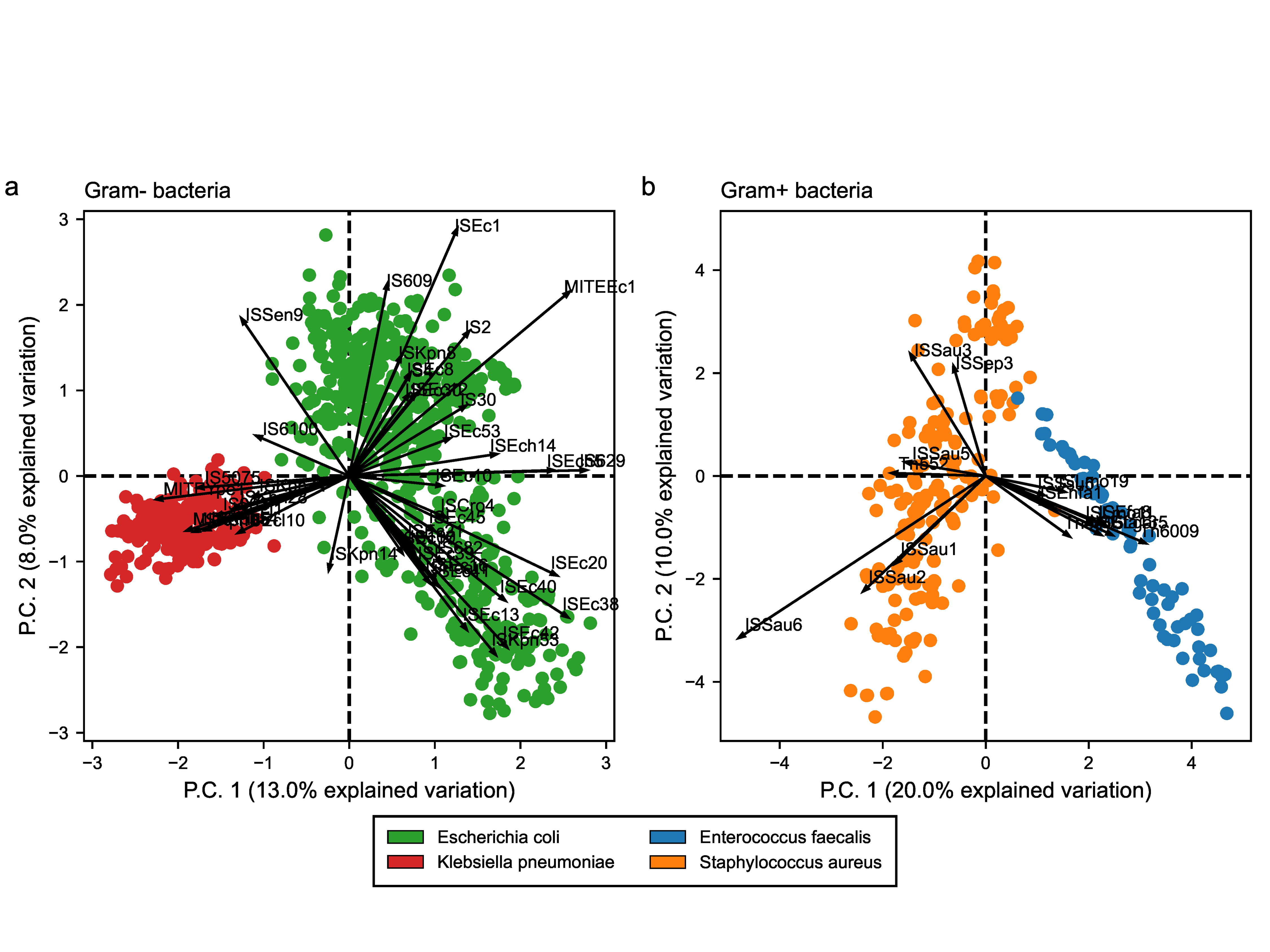

Supplement: S9 Fig — (TIF) [file pone.0330304.s013.tif]

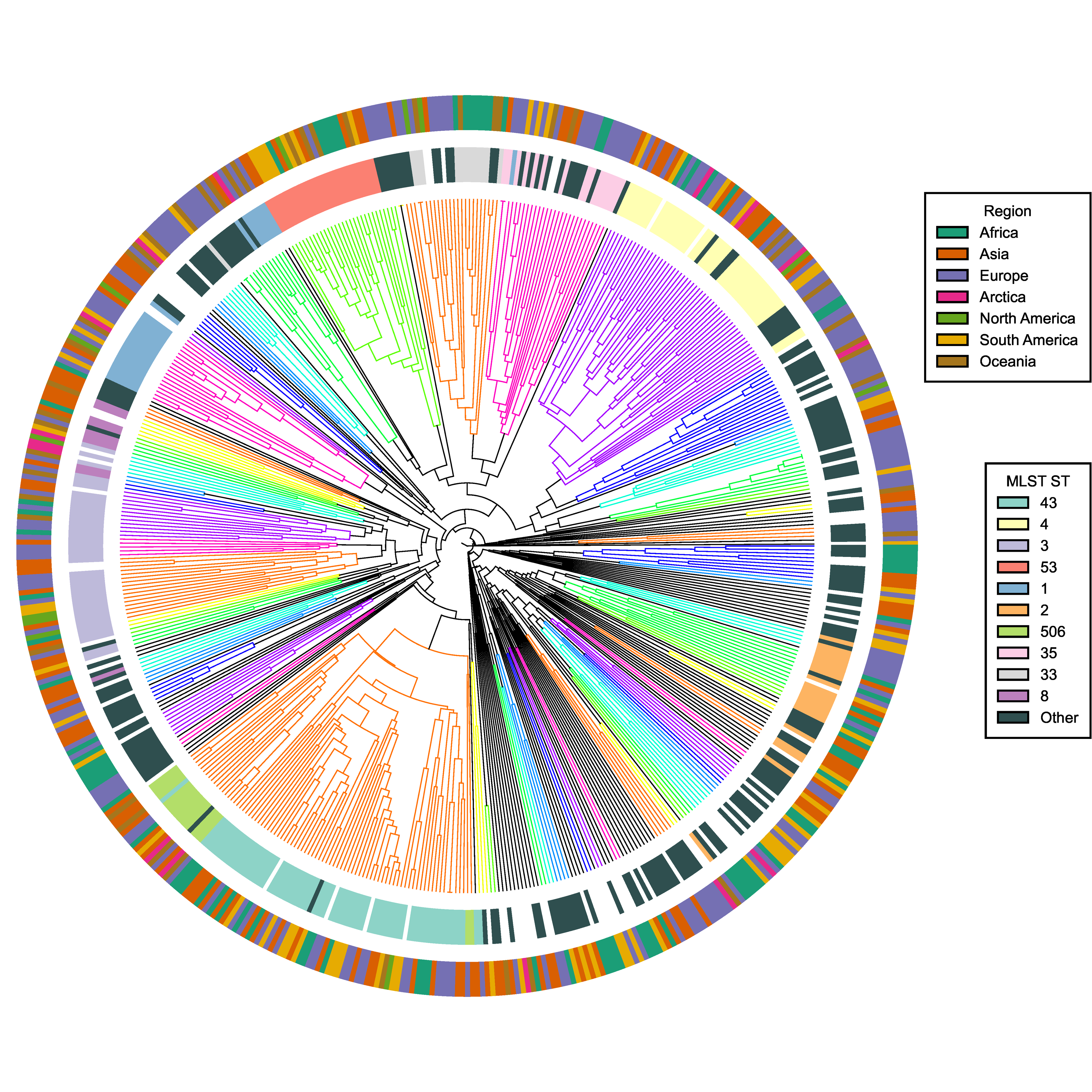

Supplement: S10 Fig — The inner circle shows MLST sequence types (ST) for the ten most frequent STs. Rare STs are amalgamated into the other category, and novel STs were omitted, showing gaps. The outer circle denotes the geographical region from which the isolate was collected. Isolates were clustered using Jaccard distance and average linkage. (TIF) [file pone.0330304.s014.tif]

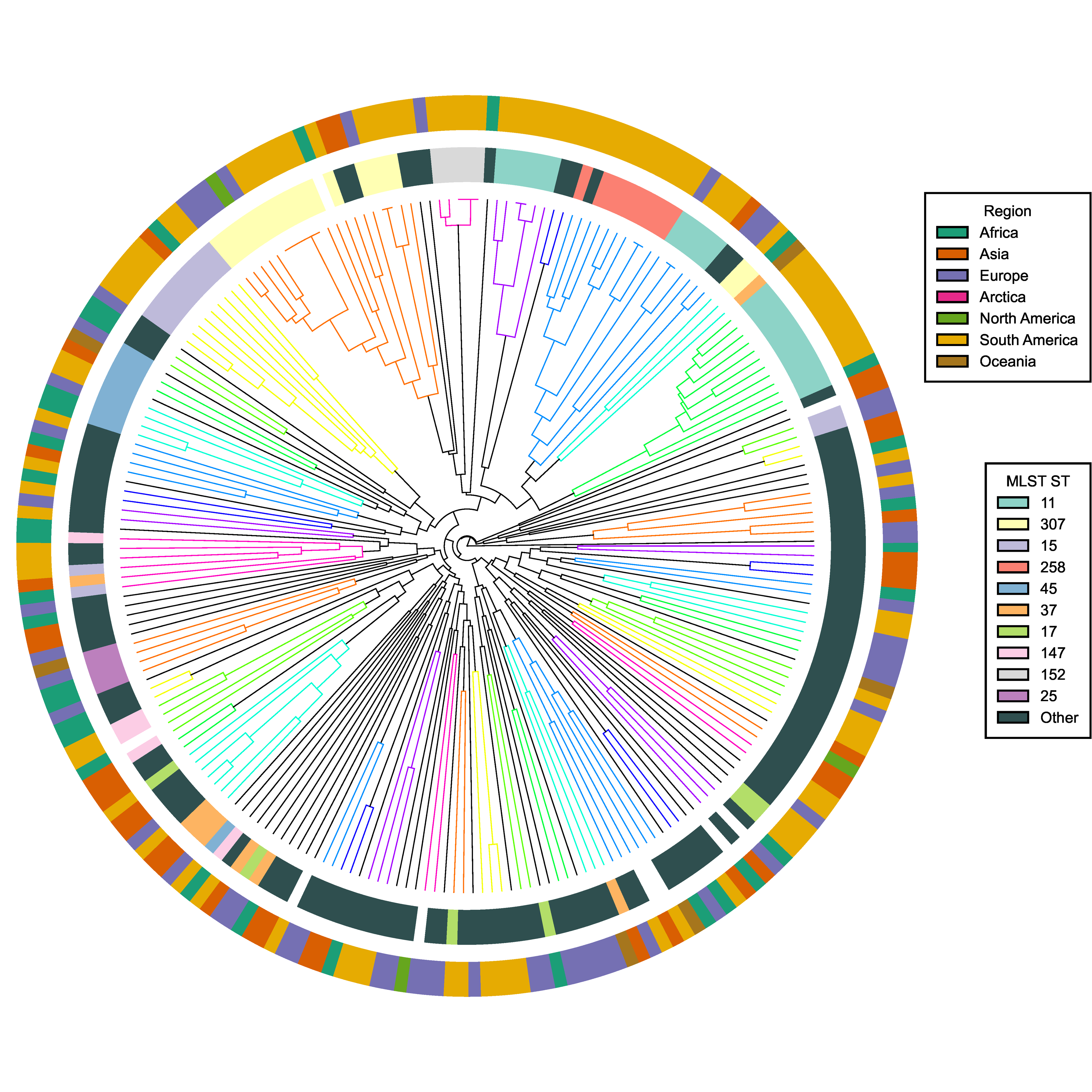

Supplement: S11 Fig — The inner circle shows MLST sequence types (ST) for the ten most frequent STs. Rare STs are amalgamated into the other category, and novel STs were omitted, showing gaps. The outer circle denotes the geographical region from which the isolate was collected. Isolates were clustered using Jaccard distance and average linkage. (TIF) [file pone.0330304.s015.tif]

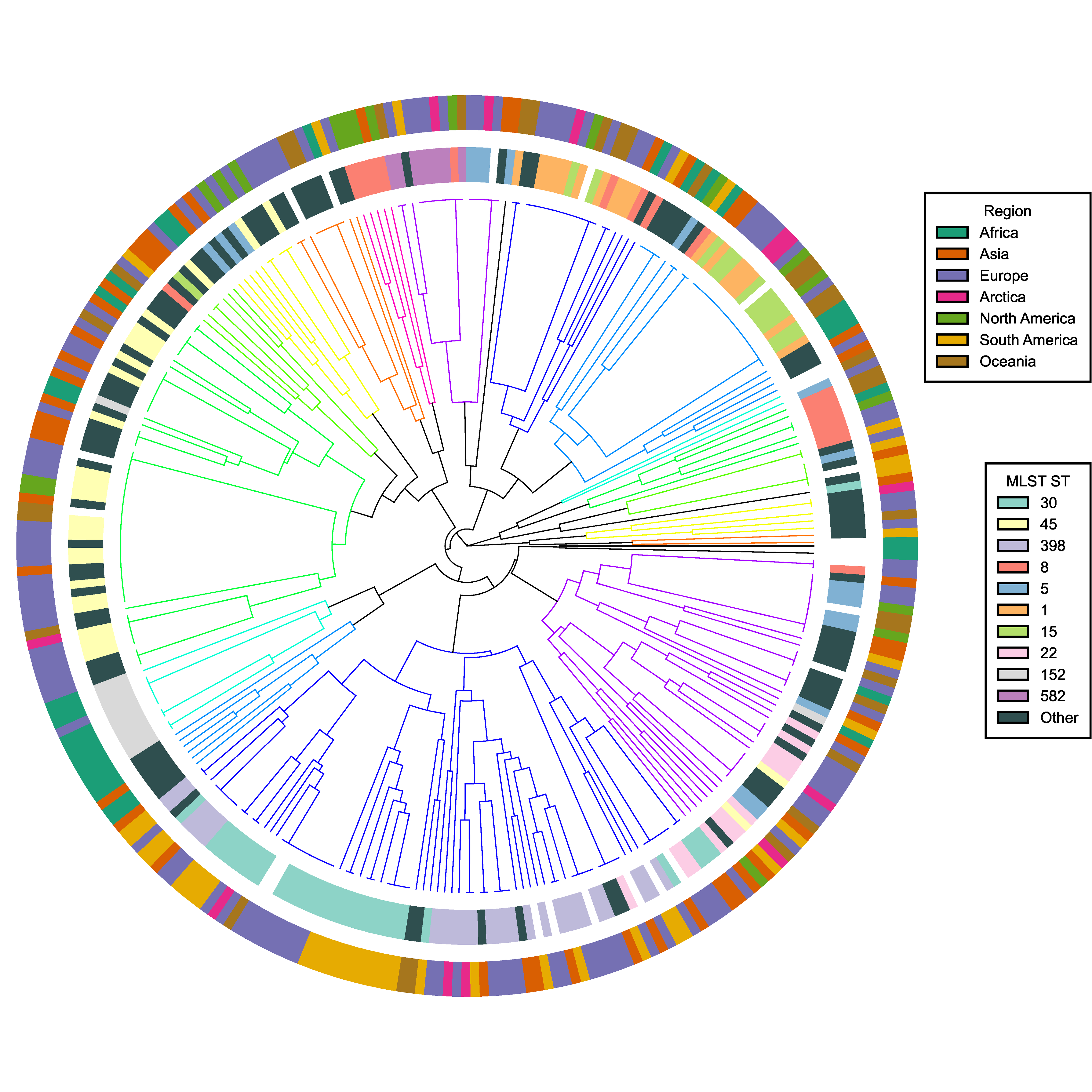

Supplement: S12 Fig — The inner circle shows MLST sequence types (ST) for the ten most frequent STs. Rare STs are amalgamated into the other category, and novel STs were omitted, showing gaps. The outer circle denotes the geographical region from which the isolate was collected. Isolates were clustered using Jaccard distance and average linkage. (TIF) [file pone.0330304.s016.tif]

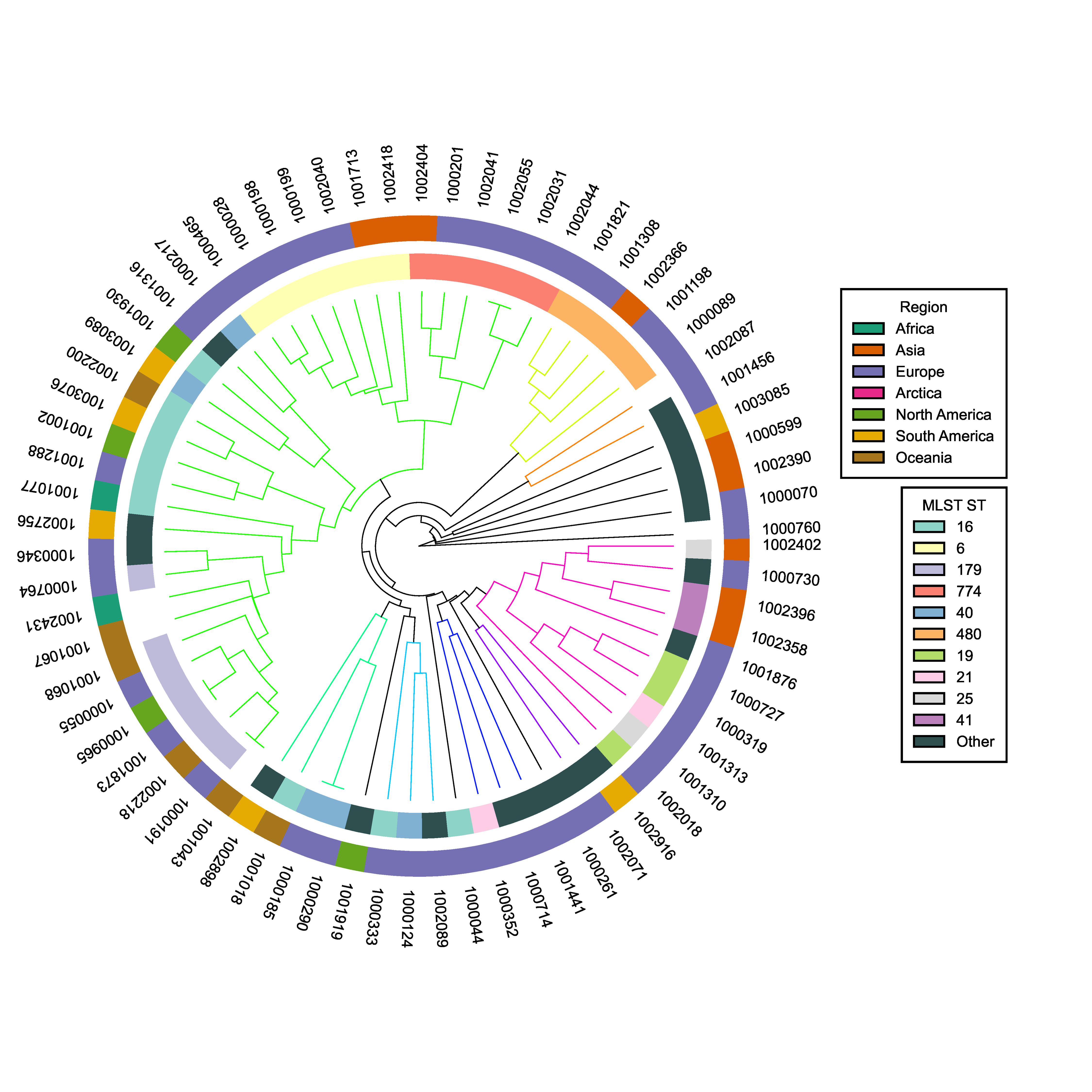

Supplement: S13 Fig — The inner circle shows MLST sequence types (ST) for the ten most frequent STs. Rare STs are amalgamated into the other category, and novel STs were omitted, showing gaps. The outer circle denotes the geographical region from which the isolate was collected. Isolates were clustered using Jaccard distance and average linkage. (TIF) [file pone.0330304.s017.tif]

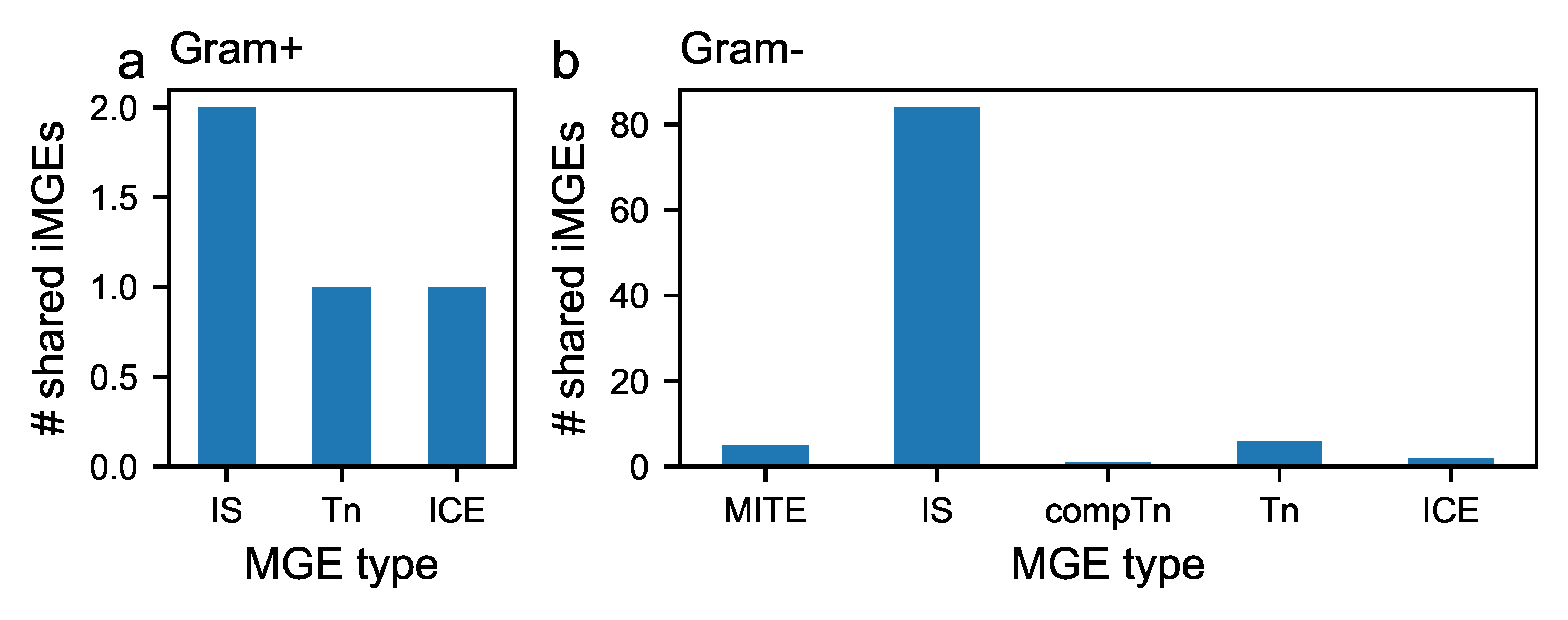

Supplement: S14 Fig — (TIF) [file pone.0330304.s018.tif]

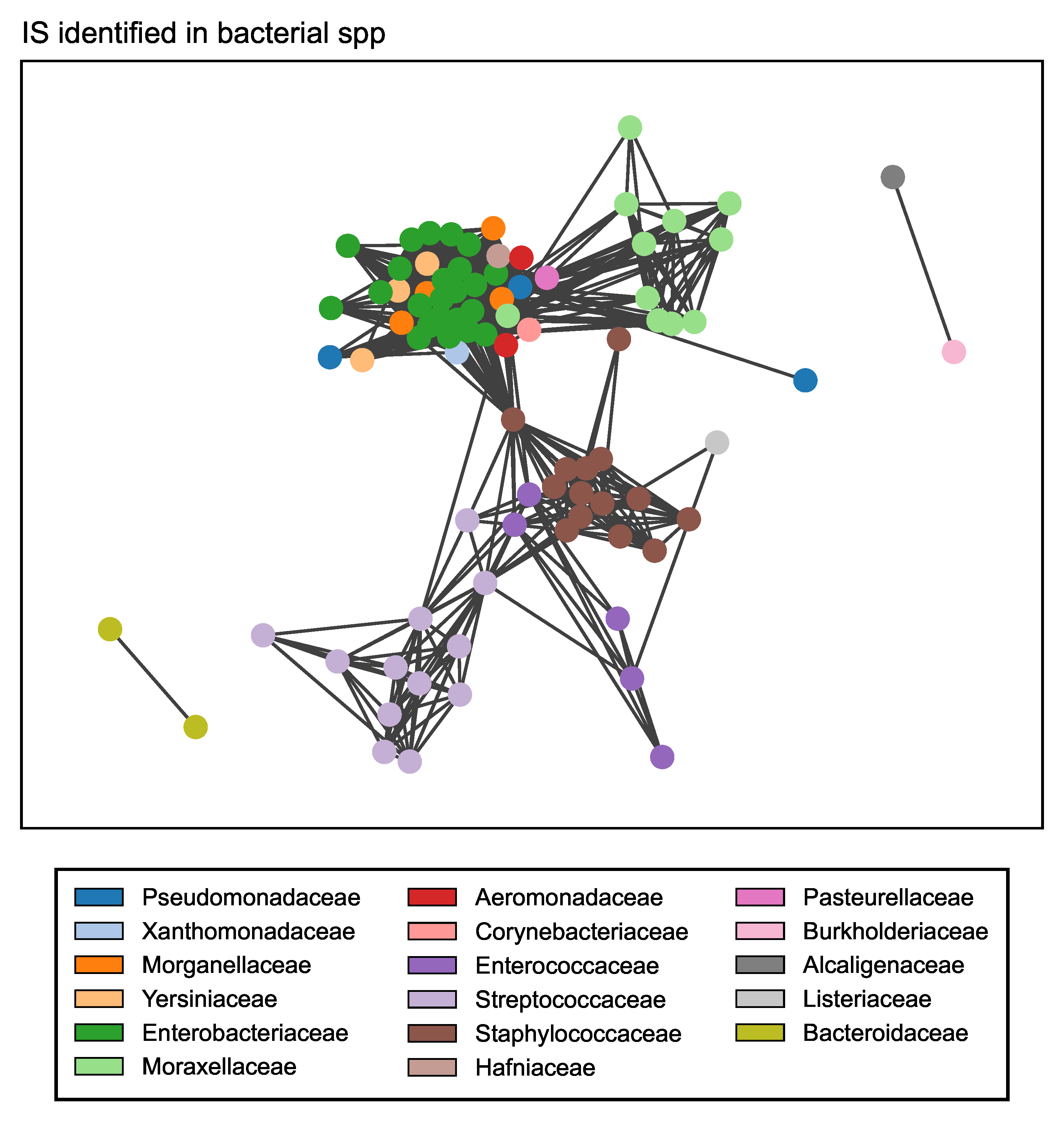

Supplement: S15 Fig — Nodes represent species and are colored by family. Edges connecting nodes represent a shared iMGE. (TIF) [file pone.0330304.s019.tif]
