# Supplementary material for: Investigation of mobile genetic elements and their association with antibiotic resistance genes in clinical pathogens worldwide
Source: PLoS One. 2025 Aug 18;20(8):e0330304. doi: 10.1371/journal.pone.0330304 (PMC12360581; doi:10.1371/journal.pone.0330304)
Supplement: S1 Table — (DOCX) [file pone.0330304.s020.docx]

Table S1. Number of beta-lactamase genes per continent in *E. coli*.

| **Family** | **Gene** | **Africa** | **Asia** | **Europe** | **North America** | **South America** | **Oceania** |
| --- | --- | --- | --- | --- | --- | --- | --- |
| **blaCMY** | blaCMY-2 | 5 | 4 | - | - | - | - |
|  | blaCMY-4 | - | 1 | - | - | - | - |
|  | blaCMY-7 | - | 1 | - | - | - | - |
|  | blaCMY-14 | - | 1 | - | - | - | - |
|  | blaCMY-15 | - | 1 | - | - | - | - |
|  | blaCMY-16 | - | 1 | - | - | - | - |
|  | blaCMY-42 | - | 5 | - | - | - | - |
|  | blaCMY-59 | - | 1 | - | - | - | - |
|  | blaCMY-131 | - | 1 | - | - | - | - |
|  | blaCMY-138 | - | 1 | - | - | - | - |
|  | blaCMY-145 | - | 1 | - | - | - | - |
|  | blaCMY-146 | - | 1 | - | - | - | - |
|  | blaCMY-149 | - | 1 | - | - | - | - |
| **blaCTX** | blaCTX-M-1 | - | - | 2 | - | - | - |
|  | blaCTX-M-2 | - | - | - | - | 1 | - |
|  | blaCTX-M-3 | - | 1 | 1 | - | - | - |
|  | blaCTX-M-14 | 1 | - | - | - | 1 | 1 |
|  | blaCTX-M-14b | - | 1 | - | - | - | 1 |
|  | blaCTX-M-15 | 37 | 41 | 20 | - | 7 | 2 |
|  | blaCTX-M-24 | - | - | - | - | - | 1 |
|  | blaCTX-M-27 | 3 | 3 | 1 | - | 3 | - |
|  | blaCTX-M-55 | - | 4 | - | - | 1 | 1 |
| **blaDHA** | blaDHA-1 | - | 8 | 1 | 1 | - | 2 |
| **blaNDM** | blaNDM-5 | 2 | 4 | - | - | - | - |
| **blaOXA** | blaOXA-1 | 26 | 26 | 15 | - | 6 | - |
|  | blaOXA-2 | - | - | 1 | - | - | - |
|  | blaOXA-10 | - | 1 | - | - | - | - |
|  | blaOXA-181 | 1 | - | - | - | - | - |
| **blaSHV** | blaSHV-1 | - | - | 3 | - | - | - |
|  | blaSHV-5 | - | 1 | - | - | - | - |
|  | blaSHV-48 | - | - | 3 | - | - | - |
|  | blaSHV-102 | - | - | 3 | - | - | - |
| **blaTEM** | blaTEM-1A | - | - | 6 | - | 3 | 1 |
|  | blaTEM-1B | 47 | 58 | 46 | 4 | 23 | 18 |
|  | blaTEM-1C | - | 1 | 8 | 1 | 2 | 1 |
|  | blaTEM-1D | 1 | - | 2 | - | - | - |
|  | blaTEM-15 | - | - | 2 | - | - | - |
|  | blaTEM-30 | - | - | 1 | - | - | - |
|  | blaTEM-34 | - | - | 1 | - | - | - |
|  | blaTEM-35 | - | 3 | 1 | - | - | - |
|  | blaTEM-36 | - | 1 | - | - | - | - |
|  | blaTEM-40 | - | - | 2 | - | - | - |
|  | blaTEM-57 | - | 1 | - | - | - | - |
|  | blaTEM-215 | 2 | - | - | - | - | - |
| **blaVEB** | blaVEB-1 | - | 1 | - | - | - | - |
|  | blaVEB-4 | - | 1 | - | - | - | - |
|  | blaVEB-5 | - | 1 | - | - | - | - |
