# Supplementary material for: Investigation of mobile genetic elements and their association with antibiotic resistance genes in clinical pathogens worldwide
Source: PLoS One. 2025 Aug 18;20(8):e0330304. doi: 10.1371/journal.pone.0330304 (PMC12360581; doi:10.1371/journal.pone.0330304)
Supplement: S2 Table — (DOCX) [file pone.0330304.s021.docx]

Table S2. Number of beta-lactamase genes per continent in *K. pneumoniae*.

| **Family** | **Gene** | **Africa** | **Asia** | **Europe** | **North America** | **South America** | **Oceania** |
| --- | --- | --- | --- | --- | --- | --- | --- |
| **blaCMY** | blaCMY-6 | - | - | 2 | - | - | - |
| **blaCTX** | blaCTX-M-1 | - | - | 1 | - | - | - |
|  | blaCTX-M-2 | - | - | - | - | 1 | - |
|  | blaCTX-M-3 | 1 | - | - | - | - | - |
|  | blaCTX-M-15 | 22 | 10 | 14 | 1 | 45 | - |
|  | blaCTX-M-55 | - | 2 | - | - | - | - |
| **blaDHA** | blaDHA-1 | - | 3 | - | - | - | 1 |
| **blaKPC** | blaKPC-2 | - | - | 1 | - | 28 | - |
|  | blaKPC-3 | - | - | 1 | - | 6 | - |
| **blaLAP** | blaLAP-2 | 4 | 2 | 1 | - | - | - |
| **blaNDM** | blaNDM-1 | 1 | 2 | 2 | - | 1 | - |
|  | blaNDM-5 | - | - | - | - | 15 | - |
| **blaOXA** | blaOXA-1 | 15 | 7 | 11 | 1 | 50 | - |
|  | blaOXA-9 | 3 | 2 | - | - | 13 | - |
|  | blaOXA-10 | 1 | - | - | - | - | 1 |
|  | blaOXA-48 | - | 1 | - | - | - | - |
|  | blaOXA-181 | 1 | - | - | - | - | - |
|  | blaOXA-232 | - | 3 | - | - | - | - |
| **blaSHV** | blaSHV-1 | - | - | 1 | - | - | - |
|  | blaSHV-11 | 4 | 7 | 3 | - | 5 | 1 |
|  | blaSHV-12 | - | - | - | - | 5 | - |
|  | blaSHV-13 | - | 3 | 1 | - | 1 | 1 |
|  | blaSHV-14 | 1 | - | - | - | - | - |
|  | blaSHV-26 | 4 | 6 | 3 | - | 5 | 1 |
|  | blaSHV-27 | 2 | 3 | 1 | - | 5 | 1 |
|  | blaSHV-28 | 6 | 2 | 11 | 1 | 20 | - |
|  | blaSHV-30 | 1 | - | - | - | - | - |
|  | blaSHV-33 | - | 4 | 4 | - | 3 | - |
|  | blaSHV-36 | - | 1 | 1 | 1 | - | - |
|  | blaSHV-38 | - | 1 | - | - | - | - |
|  | blaSHV-40 | 2 | 5 | 8 | 1 | 8 | - |
|  | blaSHV-56 | 2 | 5 | 8 | 1 | 8 | - |
|  | blaSHV-59 | - | - | - | - | 1 | - |
|  | blaSHV-62 | 1 | - | 1 | - | - | - |
|  | blaSHV-65 | 1 | - | - | - | - | - |
|  | blaSHV-67 | 3 | 3 | - | - | 3 | - |
|  | blaSHV-70 | - | 3 | 1 | - | 1 | 1 |
|  | blaSHV-74 | - | - | 1 | - | - | - |
|  | blaSHV-75 | - | 1 | 4 | - | 1 | - |
|  | blaSHV-77 | - | - | 1 | - | - | - |
|  | blaSHV-78 | 4 | 5 | 2 | - | 5 | 1 |
|  | blaSHV-79 | 2 | 5 | 8 | 1 | 8 | - |
|  | blaSHV-80 | - | 1 | 1 | - | - | - |
|  | blaSHV-81 | 4 | 3 | 2 | - | - | 1 |
|  | blaSHV-85 | 2 | 5 | 8 | 1 | 8 | - |
|  | blaSHV-86 | - | - | - | - | 2 | - |
|  | blaSHV-89 | 2 | 5 | 8 | 1 | 8 | - |
|  | blaSHV-94 | 1 | - | 2 | - | 3 | - |
|  | blaSHV-96 | 1 | - | 2 | - | 3 | - |
|  | blaSHV-98 | 4 | 5 | 2 | - | 5 | 1 |
|  | blaSHV-99 | - | - | - | - | 3 | - |
|  | blaSHV-101 | - | 2 | 3 | - | - | - |
|  | blaSHV-103 | 1 | - | - | - | - | - |
|  | blaSHV-106 | 6 | 2 | 10 | 1 | 19 | - |
|  | blaSHV-107 | - | - | 1 | - | - | - |
|  | blaSHV-108 | 1 | - | 2 | - | 1 | - |
|  | blaSHV-110 | 4 | 3 | 2 | - | 2 | 1 |
|  | blaSHV-133 | 1 | - | - | - | - | - |
|  | blaSHV-143 | - | - | 1 | - | - | - |
|  | blaSHV-145 | 4 | 5 | 2 | - | 5 | 1 |
|  | blaSHV-155 | - | - | - | - | 1 | - |
|  | blaSHV-161 | - | - | 1 | - | - | - |
|  | blaSHV-164 | - | - | - | - | 1 | - |
|  | blaSHV-172 | 1 | - | 2 | - | 5 | - |
|  | blaSHV-173 | - | - | 1 | - | - | - |
|  | blaSHV-178 | - | 1 | 1 | - | - | - |
|  | blaSHV-179 | 4 | 5 | 2 | - | 5 | 1 |
|  | blaSHV-182 | 1 | 1 | 3 | - | 22 | - |
|  | blaSHV-185 | - | 1 | 3 | - | 2 | - |
|  | blaSHV-187 | 3 | - | 3 | - | 9 | 1 |
|  | blaSHV-189 | 1 | - | - | - | - | - |
|  | blaSHV-190 | - | 1 | 2 | - | - | - |
|  | blaSHV-191 | 1 | - | - | - | 2 | - |
|  | blaSHV-193 | - | - | 1 | - | - | - |
|  | blaSHV-194 | 4 | 5 | 2 | - | 5 | 1 |
|  | blaSHV-199 | 4 | 5 | 2 | - | 5 | 1 |
| **blaTEM** | blaTEM-1A | 1 | 3 | - | - | 10 | - |
|  | blaTEM-1B | 18 | 8 | 9 | 2 | 38 | - |
|  | blaTEM-1C | - | - | - | - | 2 | - |
|  | blaTEM-30 | - | - | - | 1 | - | - |
|  | blaTEM-33 | - | - | - | - | 1 | - |
|  | blaTEM-35 | 1 | - | - | - | - | - |
|  | blaTEM-122 | - | - | - | - | 3 | - |
|  | blaTEM-163 | - | - | - | - | 3 | - |
|  | blaTEM-206 | - | - | 2 | - | - | - |
|  | blaTEM-210 | - | 1 | 1 | - | - | - |
|  | blaTEM-215 | - | - | - | - | 1 | - |
