# Supplementary material for: Investigation of mobile genetic elements and their association with antibiotic resistance genes in clinical pathogens worldwide
Source: PLoS One. 2025 Aug 18;20(8):e0330304. doi: 10.1371/journal.pone.0330304 (PMC12360581; doi:10.1371/journal.pone.0330304)
Supplement: S3 Table — (DOCX) [file pone.0330304.s022.docx]

Table S3. Statistics for two-way ANOVA testing difference in predicted plasmid content per species.

|  | Sum square | Degrees of freedom | F | P-value |
| --- | --- | --- | --- | --- |
| C(Species) | 1480.2 | 3 | 259.2 | 2.36e^-129^ |
| Residual | 2259.6 | 1187 | - | - |
