# Supplementary material for: Investigation of mobile genetic elements and their association with antibiotic resistance genes in clinical pathogens worldwide
Source: PLoS One. 2025 Aug 18;20(8):e0330304. doi: 10.1371/journal.pone.0330304 (PMC12360581; doi:10.1371/journal.pone.0330304)
Supplement: Attachment — Submitted filename: Response to Reviewers.docx [file pone.0330304.s023.docx]

Thank you for taking your time to review and provide insightful feedback on our manuscript, PONE-D-25-13754, *Investigation of mobile genetic elements and their association with antibiotic resistance genes in clinical pathogens worldwide*. We greatly appreciate your constructive advice and have incorporated many of your suggestions into our revision.

We have addressed each comment individually below and would like to highlight the following key changes to the manuscript:

- Revised the discussion to better contextualises our findings and to improve readability. It now includes a dedicated paragraph outlining the study’s limitations.
- Added a conclusion section with recommendations for future research.
- Correction of broken references and consistent use of gene nomenclature throughout the manuscript.
- A revised abstract that complies with the journal's word limit and that presents the result more specifically.

Thank you again for the opportunity to improve our manuscript. We hope the changes meet your expectations.

Sincerely,

Markus Johansson

# Detailed response to reviewers comments

## Editor

| No | Comment | Alteration |
| --- | --- | --- |
| 1 | **Limitations:** Please include a dedicated paragraph in the final part of your **Discussion** section clearly outlining the **limitations** of your study. This reflection should help contextualize your findings and guide interpretation | We added a dedicated paragraphs to discuss the limitations of the study. |
| 2 | Conclusion: Add a Conclusion section at the end of the manuscript that succinctly summarizes the main findings and their implications. | We added a conclusion section at the end of the manuscript. The conclusion outlines directions for future research. |
| 3 | Recommendations / Future Directions: Within the Conclusion section, please include recommendations derived from your key findings and/or suggest potential future research directions that could build on your work. | See comment above. |

## Reviewer 1

| No | Comment | Alteration |
| --- | --- | --- |
|  | The research topic is very interesting, and the subject is important. However, the presentation needs to be improved. The presentation of the methodologies could be improved. The findings should be well discussed because of the literature and not opinions. |  |
|  | 74 authors are too many (though there is no cut off for the number of authors for a manuscript). However, it is clear from the author's contribution that the list from lines 734-738 are the primary authors of this research. It is suggested to the authors that all other members be acknowledged for their contribution to the paper. | We share the reviewer’s concern. However, we also have to realise that collection and organising local sampling and providing the necessary epidemiological and context information is a large task. It was before initiating the study agreed with all partners that we all would be co-authors on the manuscript. |
|  | Line 122: Are 59 diagnostic units enough to represent a snapshot of clinical infections worldwide? The methodology states 35 countries. What is the justification that 59 diagnostic units from 35 countries are enough to give a clinical representation worldwide? | It is true that we have not collected data from all world countries but only a representative set of 35 countries.  We chose to describe data as a worldwide snapshot of clinical infections due to sampling from 6 world regions. We are discussing the limited number of countries in the updated discussion and have included number of countries in the abstract. |
|  | Line 142: Discussion on the identified 21 genes must be expanded | We revised and expanded the discussion of the pTUs and the associated iMGEs. |
| 1 | Line 161: Why exclude important global pathogens like Salmonella, Campylobacter, and Listeria spp. | Our intention was not to exclude important pathogens, but instead to collect a “snapshot” of bacterial pathogens encountered by the participating diagnostic units. Because the samples were not selected based on species or strains, important pathogens might only be represented by a few samples in the dataset. For example, the dataset includes one *Listeria monocytogenes* sample and 36 *Salmonella* *enterica* samples, of which 21 were from the same institute in Colombia. |
| 2 | Lines requiring citations  a. Lines 165-167 b. Lines 172-174. c. Many such sentences without appropriate citations exist in the article | Added additional citations when needed. |
| 3 | Lines 185-187: Sentence is too long | Broke up the sentence. It now reads “*The interplay of different MGEs forms a complex transposition network that has been essential for recruiting ARGs[10,20] and spreading them to infectious bacteria[21,22]. MGEs are also thought to help retain resistance genes in environments with low levels of antimicrobials.*” |
| 4 | Line 190: The collection of samples in a single year reflects a one-time period sampling, and this must be reflected in the abstract or title | We have added the collection year to the manuscript. |
| 5 | Line 191: Statement is inappropriate. Symptoms of disease caused by a pathogen are more appropriate to say rather than symptoms of a pathogen Methods | Changed the statement to say “*symptoms of a disease caused by a pathogen*” |
| 6 | Line 200: Is TWIW a name or an abbreviation? Please clarify. If it is an abbreviation, the full name should be given. All such abbreviations must be defined | Wrote out the full name of the collaboration. |
| 7 | Line 211: What comprises these seven regions is undefined | Changed to “*the 6 WHO defined regions as previously described[23]*” |
| 8 | Line 217: QC filtering may not be needed as it is deemed part of read processing | Deleted “*passed QC*” |
| 9 | Line 330: “(Error! Reference source not found)”. This must be corrected by supplying all appropriate citations. The same issue is found in other parts of the manuscript | Reviewed and corrected broken cross-references in the manuscript. |
| 10 | Lines 331-337: The nomenclature of Beta-lactamase genes must be consistent following default patterns like blaCTX-M | Corrected the formatting of beta-lactamase genes and families to follow the nomenclature. |
| 11 | Generally, the discussion must be improved to really discuss the results in the context of appropriate literature providing references | We agree with the reviewer and have reworked the discussion to increase clarity and to put our findings in context of relevant research. |
| 12 | Line 549: For abbreviation, the name should appear first, followed by the abbreviation. Example Escherichia coli (E. coli) | The abbreviations were reviewed and corrected when needed.  However, abbreviated species names were not written the first time a species name was used as it would not to conform to PLOS style guides. |
| 13 | The date of assessment of all Web pages and other documents without doi(s) must be provided in addition to the links | Dates when webpages and other online resources were accessed have been added. |

## Reviewer 2

| No | Comment | Alteration |
| --- | --- | --- |
| 1 | There are several instances of “Error reference source not found”, please review and update. | The broken references have been updated. |
| 2 | The word count of the abstract exceeded the PLOS ONE requirement of 300 words maximum. | We have shortened the abstract to 298 words |
| 3 | The authors should include the implications of this study and how it contributes towards antimicrobial resistance solutions in public health. | We agree with the reviewer and have revised the discussion to better put our findings into the context of public health. |
| 4 | Line 211: clarify what this means “and is NOT referred to as the “primary” dataset”. | It was a typo. The text has been changed to “and is now referred to as…” |
| 5 | Line 373-375: The appropriate figure for reference is Figure S8. See the Supporting information list and caption too | We have reviewed the supplementary material and updated reference and captions when needed. |
| 6 | Line 375-378: The appropriate figure to reference is Figure S9. See the Supporting information list and caption too. | See response to comment no 5. |
| 7 | The list (title and caption) for figures in the supporting information at the end of the manuscript does not match the order of their respective figures starting from Figure S3 up to S9. I will suggest a review of the figures referenced in the result section too. | See response to comment no 5. |

## Reviewer 3

| No | Comment | Alteration |
| --- | --- | --- |
| 1 | In the Abstract, results were presented more like generalized and this was seen in the Results section of the manuscript. The authors would need to be more specific in describing that results in terms of number and/or percentages with possible denominators where necessary. Using generalized terms like "Many, Most, Some" does not give a comprehensive idea of the magnitude of ARGs, iMGEs including their diversity in the species of interest.  I could see that the charts and table show some quantitative results but it will be good to include them in the results description as well as put them in context. | The abstract has been reworked to be more specific and to comply with the 300 word limit. |
| 2 | The missing references sources should to fixed by the authors. It makes the article easier to follow should the review wants to check the cited/referenced articles. | Reviewed and fixed the broken references. |
| 3 | The section on "Controlling for high relatedness in the dataset" can be moved to methods because it is not actually describing the results. It describes more of methods. Same as the opening paragraph in the results except the authors consider rephrasing them to actually mean the results if they must stay in the result section. | We agree with the reviewer’s suggestion and have moved the “Controlling for high relatedness…” to the materials and methods section.  We have rephrased the first paragraph in the results as we believe it provides the reader with some context without having to refer to the materials and methods. |
