# Supplementary material for: Investigation of mobile genetic elements and their association with antibiotic resistance genes in clinical pathogens worldwide
Source: PLoS One. 2025 Aug 18;20(8):e0330304. doi: 10.1371/journal.pone.0330304 (PMC12360581; doi:10.1371/journal.pone.0330304)
Supplement: Attachment — Submitted filename: Response_to_Reviewers_auresp_2.docx [file pone.0330304.s024.docx]

Dear Editor and Reviewers,

Thank you for the opportunity to revise our manuscript “*Investigation of mobile genetic elements and their association with antibiotic resistance genes in clinical pathogens worldwide”* (Submission ID: PONE-D-25-13754). We are grateful for the thoughtful and constructive feedback, which has helped us to improve the clarity and accuracy of our work.

We sincerely thank the reviewer for their valuable feedback and hope that the revised version meets the expectations of the reviewers and editorial team.

Sincerely,

Markus Johansson

## Reviewer 2

### Comment 1

Figure 1 – Geographic distribution of β-lactamase gene families in E. coli
The current map in Figure 1 does not include the African continent, which creates an inconsistency (compare with Figure 2) and Africa is explicitly referenced in Line 309-310. I recommend updating the figure to include Africa in order to accurately reflect the geographic scope discussed in the text.

Response:

We agree with the reviewer's observation. We have redrawn Figure 1 to include the African continent, ensuring that it reflects the data being discussed in the text and that the figure is consistent with Figure 2.

### Comment 2

Line 354 – Inconsistent Figure Referencing
The current reference style in this line — “S8 Fig S8” — appears inconsistent. I suggest the authors adopt a consistent and standard format for referencing all figures throughout the manuscript (e.g., “S8 Fig” or “Fig S8,” not both), and apply this uniformly across the document.

Response:

Thank you for pointing this out. We have carefully reviewed the manuscript and corrected all inconsistent figure references to ensure uniformity. The following changes were made:

Line 303; “S3 Fig S3b” was changed to “S3b Fig”

Line 334; “Fig S4” was changed to “S4 Fig”

Line 340; “Fig S5” was changed to “S5 Fig”

Line 341; “Fig S6” was changed to “S6 Fig”

Line 348; “Fig S7” was changed to “Fig S7”

Line 354; “S8 Fig S8” was changed to “S8 Fig”

Line 356; “Fig S9” was changed to “S9 Fig”

Line 398; “Fig S3” was changed to “S3 Fig”
